# SARS-CoV-2 and allergy – what have we learned after two and a half years? 

**DOI:** 10.5414/ALX02373E

**Published:** 2023-03-31

**Authors:** Knut Brockow, Rosi Wang, Sonja Mathes, Rebekka Bent, Valentina Faihs, Bernadette Eberlein, Ulf Darsow, Tilo Biedermann

**Keywords:** SARS-CoV-2, COVID-19, allergy, vaccination, drug reaction, skin reaction, anaphylaxis, allergy tests, polyethylene glycol, polysorbate 80

## Abstract

Background: Coronavirus disease-2019 (COVID-19) has significantly hampered the regular workflow for allergists and allergy departments. Materials and methods: The purpose of this review is to highlight our own experiences on SARS-CoV-2 and allergy as well as to discuss findings from the literature. Results: Vaccination against SARS-CoV-2 is needed for protection against severe infection. Skin reactions may arise with SARS-CoV-2 infections. Short-term general immune reactions and skin reactions are also possible upon SARS-CoV-2 vaccination; however, they recur in only a proportion of patients during follow-up vaccinations. Initial reports of anaphylaxis after vaccination fueled public fear. On the other hand, more recent epidemiologic data do not show a substantially increased anaphylaxis risk compared with other vaccines. Fear-related reactions may be essential for many “anaphylaxis” reports. In Germany, the flow chart developed by Paul-Ehrlich-Institut (PEI) and Robert-Koch-Institut (RKI) together with the allergological societies helps to care for patients with suspected “allergy history” safely and effectively. Through this, patients with increased risk of anaphylaxis to SARS-CoV-2 vaccines and their ingredients (e.g., polyethylene glycol (PEG), polysorbate 80) are identified. However, since only small amounts of these excipients are contained in mRNA vaccines, even some PEG-allergic patients can tolerate the vaccination. In Germany, an allergy test-guided procedure is recommended for high-risk patients, including an allergy history, prick tests, intradermal and basophil activation tests, and, if necessary, provocation tests. This also appears effective for anxiety reduction in patients with vaccination skepticism. To date, all of our patients have been able to be vaccinated with SARS-CoV-2 vaccines without the occurrence of significant reactions. Conclusion: Many initial concerns about unexpected side effects of SARS-CoV-2 vaccination have not been confirmed. The flowchart and, in the case of suspicion of hypersensitivity, an allergy test-guided risk assessment helps to reduce patients’ fear of vaccination and enables safe vaccination.

## Introduction 

The number of SARS-CoV-2 infections continued to reach new highs until autumn 2022 and has fallen since in Western countries [1]. The last wave of infections unexpectedly happened in summer 2022. The omicron variants have now successively displaced all other SARS-CoV-2 variants. They show higher infectivity compared to the previous variants. Fortunately, illness with an omicron variant is milder and results in fewer deaths and a lower burden on the healthcare system through hospitalization and intensive care bed occupancy. With baseline vaccination of over 76% and booster vaccinations of over 61% of the total population, the majority of the population is protected from severe complications [1]. Protection is reduced in at-risk patients with compromised immune systems. Deaths from SARS-CoV-2 infection now largely affect elderly patients, patients with immunosuppression, and those with severe pre-existing conditions. For these patients, the removal of measures such as mandatory mask carriage requirements pose an additional risk. The effectiveness of SARS-CoV-2 vaccination against getting an infection with an omicron variant is reduced compared with that to earlier variants. There is a decrease in the efficacy of the basic immunization after 2 – 3 months, especially in patients with impaired immune systems. Therefore, a booster vaccination is particularly important for these patients. 

In this respect, vaccination is important and necessary to combat the SARS-CoV-2 pandemic. However, on the other hand, there is public fear of possible side effects, especially allergic reactions to SARS-CoV-2 vaccination [2]. Already on the first day of the vaccination campaign in the UK with a novel mRNA vaccine, anaphylaxis after vaccination was reported [3, 4]. A possible danger for “allergy sufferers” was presented and spread by the media. As a result, vaccinating physicians, allergists, and allergy departments were contacted on a large scale by patients who were afraid or skeptical of vaccination and also by unsure medical colleagues. Our allergy department also had to try as best as possible to handle the multiple inquiries without neglecting the other allergy patients. This was difficult without additional financial support. In addition, there was initially no experience on the anaphylaxis risk from SARS-CoV-2 vaccines. 

The purpose of this article is to provide an overview of hypersensitivity reactions in the setting of COVID-19 infections, of expected vaccine side effects, of allergy diagnostic measures used for suspected vaccine reactions, and of new evidence that explains such reactions and which has led to changes in the management and diagnosis of such reactions. 

## Hypersensitivity reactions to drugs used to combat SARS-CoV-2 infection 

Already a few months after the start of the COVID-19 pandemic, the EAACI Drug Allergy Working Group analyzed the risk of taking drugs commonly used (off-label) for the treatment of COVID-19 infection for the occurrence of typical drug reactions, for example, the clinical pictures of maculopapular exanthema or urticaria [5]. Currently, antiviral antibodies, intra- and extracellularly active antiviral drugs such as remdesivir, nirmatrelvir/ritonavir, or, in severe inflammatory responses, dexamethasone and other immunosuppressants such as tocilizumab, anakinra, or baricitinib and intravenous immunoglobulins are used [5, 6]. At the beginning of the pandemic, convalescent plasma, ivermectin, colchicine, chloroquine, azithromycin, interferon-alpha, and heparin were also used without any benefit to patients [5, 6]. However, all these drugs are not common triggers of drug allergies. Drug exanthema to antibiotics should be considered in all infections. These may be used secondarily to treat complications of COVID-19, such as beta-lactams (for example, amoxicillin) for secondary bacterial infections [5, 7]. 

## Skin manifestations in SARS-CoV-2 infection 

Much more frequently, in our own patients and in the literature, we find skin manifestations during or after infections with SARS-CoV-2 itself, rather than to drugs used to treat COVID-19 [8]. We performed a literature review and found prevalence data of such skin manifestations among COVID-19-infected patients ranging from 0.2 to 20% [9]. The most common manifestations were maculopapular exanthema, papulovesicular exanthema, acute urticaria, and vascular reactions [8]. Among the latter, pernio-like acral lesions were particularly common in children who had good general health and in whom SARS-CoV-2 infection was undetectable in the vast majority, but who had close contact with infected persons [10, 11]. In addition, livedoid and purpuric lesions in COVID-19 have been described as relatively specific for this disease [8, 9]. Critically ill patients with SARS-CoV-2 infection also present with necrotizing acral vasculitis with microangiopathy and thrombosis, up to disseminated intravascular coagulation and high mortality [8]. Outbreaks of Kawasaki disease exanthema have been observed in children in COVID-19-endemic areas [12]. 

## General side effects to be expected after vaccination against SARS-CoV-2 

**Figure 1.**
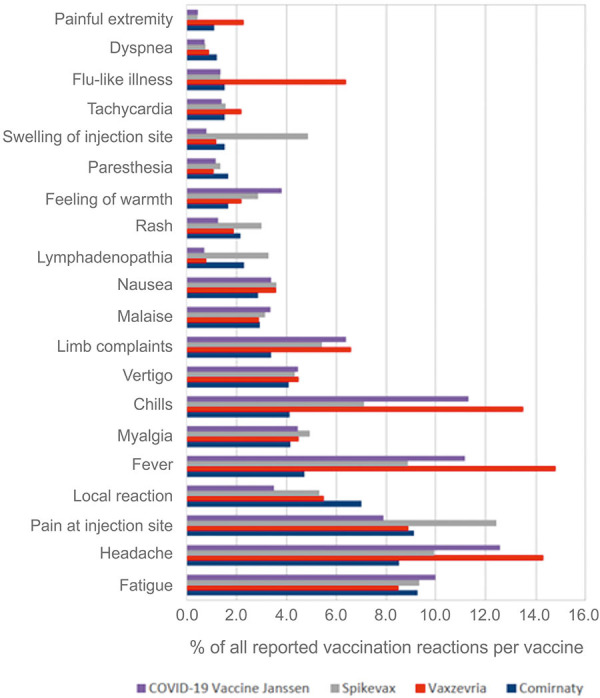
Adverse reactions frequently reported to the Paul Ehrlich Institute after vaccination with Spikevax (Moderna), Vaxzevria (AstraZeneca), Comirnaty (BioNTech/Pfizer), and the COVID-19 vaccine Janssen. Percent frequencies are given (percentage of the number of adverse reactions reported in each case out of the total number of adverse reactions reported after administration of each COVID-19 vaccine). Source: https://www.pei.de/DE/newsroom/dossier/coronavirus/coronavirus-inhalt.html, Safety-Report-27-12-to-31-07-21.

General side effects can be expected with all active vaccinations, with varying frequency and severity. Active vaccines basically act via specific activation of the immune system against virus-infected cells. The available SARS-CoV-2 mRNA vaccines also act via expression of viral proteins, particularly the spike protein in host cells. Cells of the innate immune system are activated and produce, among other things, type 1 interferons, other cytokines, and chemokines, which initiate a cascade that leads to the formation of specific effector T cells and later memory T cells. CD4^+^ effector T cells, the T helper cells, are in turn required for antibody production by B cells against viral components [13]. This process, which is started by vaccination, is overall very specific against the virus. Meanwhile, bystander cells are activated, and many proinflammatory signals take effect, so that unspecific vaccine reactions may also be observed in some patients. These usually last only a few days and often present as flu-like symptoms (Figure 1) with fever, fatigue, exhaustion, and possibly chills, headache, muscle, limb, and joint pain, nausea, tachycardia, and lymphadenopathy [14]. In addition, local vaccination reactions with pain, swelling, and redness at the vaccination site several hours to 3 days after vaccination are relatively common [14]. Delayed local vaccination reactions also may occur up to 2 weeks after vaccination [15]. 

## Skin reactions after vaccination against SARS-CoV-2 

Vaccinations, like infections, can trigger skin reactions. We observed a number of skin reactions after SARS-CoV-2 vaccination in our clinic [16, 17]. These included in particular urticaria, angioedema, and flush a few hours after vaccination, but also delayed hypersensitivity reactions such as local injection reactions or exanthema that developed after several hours to days. In predisposed patients, vaccination triggered chronic inflammatory skin diseases, for example, eczema and psoriasis. In others, because of endogenous virus reactivation probably associated with a post-vaccination short-lasting immune deficit against these viruses, pityriasis rosea or zoster infections developed. Other dermatological manifestations were less common. Our observations correspond well with those reported in the literature [18]. Again, immediate as well as delayed local reactions, urticaria, and morbilliform exanthema have been described in particular, occurring a few days after vaccination and usually resolving within a week thereafter [18]. The incidence of such skin reactions after SARS-CoV-2 vaccination is not known. Even if there is a temporal relationship between vaccination and skin manifestation, not all of these reactions must also be causally related with the vaccine. Causality appears to be confirmed in those patients who have the same objective reaction when vaccinated again. When the same vaccine was given again, we found recurring skin reactions in a bit less than 50%, which has also been reported in the literature [18]. Such reactions should not normally lead to not administering the next vaccination. In patients with urticaria after vaccination, we performed exemplary allergy tests, all of which were negative. 

## Severe complications of vaccination against SARS-CoV-2 

In total, more than 12 billion vaccine doses have been given worldwide, and more than 180 million vaccinations have been performed in Germany [19]. Regarding severe complications, SARS-CoV-2 vaccination shows a high safety. The following safety signals were found after application of SARS-CoV-2 vaccines: after mRNA vaccination a higher number of myocarditis and pericarditis were found than would be expected [20]. These were mild in most cases and mostly not a contraindication to revaccination. After SARS-CoV-2 vector vaccination, there was a signal of increased risk of thrombosis at atypical sites including cerebral thrombosis 5 – 10 days after vaccination [21]. Patients under 60 years of age were particularly affected at a rate of ~ 10 cases per million vaccinations. In some of these patients, vaccine-induced immunologic thrombotic thrombocytopenia with antibodies against platelet factor 4 protein, which can activate platelets via the formation of immune complexes, has been demonstrated [21]. Patients with such vaccine-induced thromboses may not receive the SARS-CoV-2 vector vaccine in the future (vaccination with mRNA vaccines or Nuvaxovid, Novavax, Gaithersburg, MD, USA, is possible in these cases). In addition, after the administration of SARS-CoV-2 vector vaccines, there was a questionable signal for the occurrence of Guillain-Barré syndrome; thus, patients with a history of such syndrome should avoid SARS-CoV-2 vector vaccines [22]. The vast majority of patients with immune-mediated inflammatory diseases tolerate SARS-CoV-2 vaccination well [23]. Concerns about worsening of disease after vaccination are usually not confirmed. An individual benefit/risk assessment is positive in the vast majority of patients. In this respect, there are no substantial signals for a vaccination-induced worsening and no contraindication to vaccination for the vast majority of patients with autoimmune disease (with the exception of idiopathic thrombocytopenic purpura for the vector vaccine Vaxzevria) [23]. 

## Anaphylaxis after SARS-CoV-2 vaccination 

Anaphylaxis, as the most severe form of immediate allergic reaction, is life-threatening. In this respect, the fear of anaphylaxis caused by novel mRNA SARS-CoV-2 vaccines was great. Approval studies showed no risk signals, and the vaccines were approved. However, cases of anaphylaxis were reported on the very first day of the vaccination campaign in the United Kingdom. Similarly, for all other SARS-CoV-2 vaccines, incidences of anaphylaxis were shown to be increased in initial surveys compared with other vaccines (for example, influenza vaccination) [3, 24]. 

Because SARS-CoV-2 vaccines are used for large-scale population vaccination and mRNA vaccines have not been previously used before, these reactions received widespread media interest and dissemination. They led to considerable uncertainty in the population. Both, patients and physicians were concerned and searched for causes of the initially significantly increased incidences reported. Of all the ingredients, the excipients polyethylene glycol (PEG) and polysorbate 80 (PS80) were suspect, as allergic reactions to these substances had previously been described. 

Meanwhile, incidence rates in Germany for anaphylaxis to SARS-CoV-2 vaccines marketed in Germany have been less than five cases per million based on anaphylaxis reports to the Paul Ehrlich Institute (PEI) through the end of 2021. This rate was discussed in a systematic review to be not or only slightly elevated compared with the average for other vaccines of about 1.3 cases per million vaccinations [25]. Women are significantly more frequently affected in all surveys, especially women with pre-existing allergies or anaphylaxis [3, 25, 26, 27]. Most reactions occur to the first dose of a vaccine. There is a negative trend in all reports of anaphylaxis over time since approval indicating possible reporting bias, and there have been no published deaths from anaphylaxis related to vaccination that we are aware of. 

## Allergy testing – controlled procedure before vaccination in Europe 

### Indication for allergy testing 

**Figure 2.**
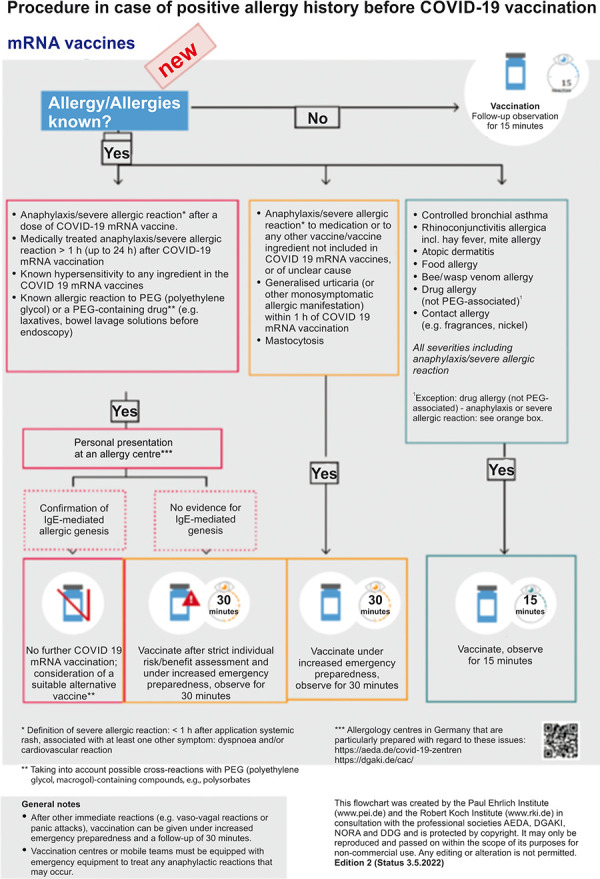
Updated flowchart on the procedure in patients with positive allergy history before COVID-19 vaccination.

The PEI and RKI in collaboration with the allergological societies DGAKI and AEDA (participation as experts: Margitta Worm, Ludger Klimek, Knut Brockow) developed a flow chart on the recommended procedure in case of positive allergy history before SARS-CoV-2 vaccination [26], which helps to determine the indication for allergy testing and is now published in a new edition (Figure 2). Only if there is evidence of anaphylaxis to previous doses of a SARS-CoV-2 vaccine or known hypersensitivity to an ingredient in the vaccines, particularly PEG, a prior allergy evaluation is indicated. All other patients can be vaccinated directly. Already in early 2020, we from the European Competence Network Mastocytosis advocated to vaccinate patients with mastocytosis [28]. Meanwhile it has been demonstrated that patients with mastocytosis do not have a general increased risk of adverse reactions to vaccination [29]. In patients with onset of generalized urticaria after SARS-CoV-2 vaccination or with a history of anaphylaxis to other vaccines, vaccination should be given under emergency preparedness and with extended monitoring time. 

### Possible triggers of anaphylaxis 

As possible triggers of anaphylaxis to SARS-CoV-2 vaccines, initially mainly PEG and PS80 were discussed. Case series with severe allergies and positive skin tests have already been well described for PEG, which is used as a binding excipient not only as laxatives but also in many medical injection solutions and tablets, juices, suppositories, and other forms of application as well as cosmetics used at home [30]. However, the number of patients with reported anaphylaxis to SARS-CoV-2 vaccine is many times higher than the total number of PEG-allergic patients reported in the literature (< 300 patients in the last 30 years, according to our research) [31]. PS80 and other derivatives of PEG could theoretically cause cross-reactions. However, clinically relevant cross-reactions have been very rarely shown in PEG-allergic patients, even if some skin tests were positive for PS80 in these patients [32]. PS80 is not only used in vector and protein vaccines against SARS-CoV-2, but also in many biologicals. A causative role of PS80 for reactions to these biologicals has been discussed, but has only been adequately demonstrated in very few individual cases. The role of PS80 as a trigger of anaphylaxis to SARS-CoV-2 vaccines remains insufficiently documented. For a possible relevance of other ingredients in SARS-CoV-2 vaccines, for example trometamol, there is insufficient evidence so far with few questionable individual case reports that are difficult to interpret, despite very high awareness. Meanwhile, we can estimate that the many described anaphylactoid reactions to mRNA vaccines cannot be explained by the few cases of revealed PEG allergy or allergy to another excipient. Rather, we speculate that anxiety and stress reactions play a significant role in many patients and that many reactions may not represent true anaphylaxis, but stress reactions or vasovagal syncope. 

### Vaccination recommendations after allergy testing 

In one paper, we summarized our cases of allergy assessment and SARS-CoV-2 vaccine tolerability in 421 patients with pre-vaccination concerns and 10 patients with proven PEG allergy [33]. Skin testing with prick test and intradermal test and confirmation by basophil activation test in patients with evidence of PEG allergy were used to evaluate these patients for the presence of PEG allergy. In 94% of patients with concerns about vaccine safety, no increased risk was demonstrated, and routine vaccination was recommended. Twelve additional patients showed negative allergy test results; however, because of the severity and timing of the reaction, nonallergic hypersensitivity could not be ruled out with certainty. These patients also tolerated vaccination under individualized precautions with longer observation periods (n = 12), premedication with antihistamines (n = 4), fractionated vaccine administration (n = 1), or inpatient vaccination (n = 1). Three further patients had a new initial diagnosis of PEG allergy. Two of these patients under monitoring underwent immunization with (DNA) vector vaccines (Vaxzevria) without reactions, 1 patient refused vaccination. Of the 10 PEG-allergic patients, some of whom had been diagnosed many years earlier, tolerability information could be obtained from 4 patients: 2 patients tolerated vaccination with (DNA) vector vaccines (Vaxzevria), 1 with mRNA Comirnaty after prophylactic H1 antihistamine administration and 1 the initial vaccination with Vaxzevria and booster with Comirnaty. In summary, all allergy-tested patients tolerated the performed SARS-CoV-2 vaccination without significant side effects. 

A similar approach is taken in several allergy testing centers in Europe [26, 34, 35, 36]. Patients with allergy history for possible SARS-CoV-2 vaccine hypersensitivity and patients with PEG allergy were tested. A specific recommendation for vaccination was made depending on the result of the allergy test. Allergy testing protocols vary depending on the availability of test substances and experience with test methods. Thus, testing takes place with the vaccines themselves (if available) as well as with the pure adjuvants (available in some centers in Europe) or with the adjuvants in drugs (in the USA). Skin prick tests, intradermal tests, basophil activation tests and provocation tests may be used. Systemic reactions occurred in individual patients with PEG allergy during skin testing. In the previous reports of successful vaccination, there was no report of a severe reaction after allergy testing was performed and recommendations were followed. 

## Tolerance despite reaction to the first vaccine dose 

In the USA, a different approach is often taken without prior allergy testing. Patients who did not experience an excessive anaphylactic reaction were revaccinated under supervision [25, 27, 37]. For example, in one study, 189 patients with reactions to the first vaccination were evaluated for tolerance of another vaccination [37]. Of these, 159 patients, including 19 patients with previous anaphylaxis, received a second dose. Antihistamine premedication was administered to 30% of patients. Of these patients, 80% had no reaction to the second dose and 20% had only mild reactions that could be treated with antihistamines. These results rather argue against IgE-mediated allergy in most of these patients, even though an alternative hypothesis is that the amount of PEG in mRNA vaccines is much lower than in other preparations and might not reach the reaction threshold. Likewise, there are studies with patients who had a reaction to substitution therapy with PEG-asparaginase (containing PEG 5000), but had no reaction to mRNA vaccination, and some of them had previously tolerated PEG-containing laxatives [38]. 

Furthermore, reports of PEG-allergic patients who tolerated mRNA SARS-CoV-2 vaccines are increasing [33, 37]. In a patient population of 12 PEG-allergic patients, in whom PEG allergy was detected in 6 patients by positive skin or oral challenge test, no reaction occurred after vaccination with mRNA SARS-CoV-2 vaccine [39]. Furthermore, there are several case reports of patients with immediate-type PEG allergy who tolerated their vaccination [40]. These reports could mean that the small amount of PEG 2000 in vaccines is not sufficient to trigger symptoms in some PEG-allergic patients. Alternatively, reactions in many patients might not be mediated by IgE and triggered by PEG. We now believe that stress-induced reactions and vasovagal syncope without evidence of an immunologic event also play a significant role in many patients. Immunologists have also postulated a complement-mediated mechanism (complement activation-related pseudoallergy (CARPA)). In this mechanism, IgG or IgM antibodies against PEG are thought to activate complement factors and thus generate the anaphylatoxins C3a, C4a, and C5a, which could lead to mast cell degranulation. In a case series of 22 patients with suspected SARS-CoV-2 vaccine reactions, positive reactions in the basophil activation test to PEG and IgG to PEG were detected in 10 of 11 patients who were examined further. Because skin tests and IgE to PEG were negative in these patients, CARPA was suggested by the authors as a possible mechanism [41]. However, the evidence for such an explanation remains unconvincing [24, 27]. Other authors have also shown that in such patients, positive responses in the BAT to PEG or mRNA SARS-CoV-2 vaccine could be inhibited by wortmannin, indicating an IgE-mediated response [42]. The BAT has been used to detect sensitization, particularly to the mRNA SARS-CoV-2 vaccines, and to identify PEG-free alternative vaccines that were negative in the BAT [43]. 

## Allergy workup before SARS-CoV-2 vaccination 

Allergy departments worldwide were inundated with patients who had concerns about allergic reactions to SARS-CoV-2 vaccination, often medically unfounded. Waiting lists were created, and resources for these patients competed with those for patients with other allergy concerns. In our center, as in all allergy centers in Europe, allergy tests attempt to screen for PEG allergy in patients with reactions to vaccination. It was initially assumed that the majority of anaphylaxis would be triggered by PEG. We organized a SARS-CoV-2 vaccine testing week for patients with justified or unjustified fear of vaccination; to exclude possible PEG allergy, the history was taken and skin prick tests done at 30-minute intervals [44]. We were able to show that the increasing number of appointment requests in an allergy department could be handled efficiently and promptly in this way, that fears were relieved, and most patients subsequently had themselves vaccinated without developing a reaction. Our testing now has two different objectives. The first is to investigate whether allergies to ingredients are present in the patients asking for clarification, and the other is to alleviate concerns about severe allergic reactions in patients with fears about vaccination. We could show that patients not daring to get vaccinated paradoxically have more anxiety towards vaccination than to acquire COVID-19. For those, allergy testing was an effective tool to increase vaccination willingness and thereby to combat vaccination hesitancy [45]. 

In our opinion, the flowchart for the procedure of COVID-19 vaccination in case of positive allergy history [26] has proven itself in the past years and by consensus of all above mentioned parties required only minimal adjustments in the course based on the new knowledge gained in the meantime (Figure 2). Patients with suspected PEG allergy are identified by the medical history. Questions are asked about previous anaphylaxis occurring within 1 hour either after vaccination for SARS-CoV-2 or after PEG contact with a typical trigger such as laxatives (for example, Movicol), depot cortisone preparations, hormone injections, cough suppressants, or vaginal suppositories. In addition, multiple severe systemic physician-documented reactions to multiple unrelated drugs or to only some preparations of the same active ingredient may indicate a PEG allergy. An indication of good tolerance to PEG is the ingestion of laxatives containing PEG (for example, Movicol) without reaction; in these cases, no further diagnostics is necessary. These patients may also receive mRNA SARS-CoV-2 vaccines. Contact sensitization to PEG or delayed reactions after several hours are not indicative of immediate-type PEG allergy (Table 1). We have advised patients with contact sensitization to PEG to vaccinate also with mRNA vaccine and have received several responses of good tolerability from the patients, because the small amount of PEG in the vaccine usually does not elicit a reaction. 

In patients with a suspicion of possible vaccine hypersensitivity or PEG allergy, we carry out skin prick tests with vaccine residues as well as the essential adjuvants PEG, PS 80, and trometamol. According to recent information, testing PEG of higher molecular weight (e.g., PEG 20000 instead of PEG 2000) seems to have a better and sufficient sensitivity [30]. Therefore, intradermal tests, which we used to perform as a standard procedure before, only need to be performed if the skin prick test is negative and there is a strong suspicion. Intradermal tests with PEG, like the provocation test, were associated with a higher risk of systemic test reactions [46]. We found systemic reactions in our PEG-allergic patients to extremely variable thresholds, ranging from minute allergen doses inserted by skin prick test in very few patients to doses applied during intradermal test to oral administration of 38 g PEG 3350. This risk has to be taken into account in the diagnostic process, and emergency preparedness was always assured. In addition, we perform a basophil activation test in cases of suspected PEG allergy, which has no risk of systemic reactions [43]. We were able to show in a literature review that vaccines and modified PEGs (for example, PEG 2000 dimyristoyl glycerol) have a higher sensitivity when used in the basophil activation test compared to unmodified PEGs [43]. 

Although testing of culprit vaccines as such in skin test and BAT seems to be of higher importance, we continue to complement these tests with PEG and PS 80 to identify patients with allergies to these excipients. Provocation tests are the gold standard for detecting hypersensitivity in allergology. This can be carried out with PEG of different molecular weights [33, 46]. Vaccination with a suspected vaccine also corresponds to a provocation test. Depending on the individual history, vaccines can be given directly or after premedication with antihistamines, in fractionated doses, and under inpatient monitoring. Emergency preparedness must always be maintained, the patient must be adequately monitored, and emergency medications must be ready. In patients at very high risk, administration of alternative SARS-CoV-2 vaccines is possible but usually not necessary. An alternative to basic immunization against SARS-CoV-2 by RNA and vector vaccines is now available, the protein-based vaccine Nuvaxovid for persons 18 years of age and older, which has demonstrated 90% efficacy in pivotal trials. This vaccine does not contain PEG but PS 80 as well as saponins as immunostimulatory effect enhancers. Although a high level of vaccine skepticism against mRNA and vector vaccines is still reported among parts of the population, demand for the new protein-based vaccine is unexpectedly low. 

## Conclusion 

Two and a half years after introduction of the SARS-CoV-2 vaccine, many initial concerns about side effects, particularly the high risk for inducing anaphylaxis, have not been confirmed. Reaction rates are now reported to be comparable to other vaccines. The general immune reactions already known from other vaccinations and also skin reactions are possible after SARS-CoV-2 vaccination and recur in only a proportion of patients when vaccinated again. PEG allergy does not appear to be the main cause of anaphylactoid reactions reported after vaccination. Vaccination of patients with PEG allergy also appears possible with mRNA vaccines if appropriate precautions are taken. In Germany, the flowchart on the procedure of COVID-19 vaccination for patients with a positive history of allergy provides a feasible tool to care for patients safely and effectively. For patients with suspected SARS-CoV-2 vaccine hypersensitivity or PEG allergy, an allergy test-guided procedure continues to be recommended in Europe, in which an allergy history, skin prick test, optional intradermal test, basophil activation testing, and provocation test may be used. Allergy testing has also appeared to be effective in reducing anxiety in patients with vaccine skepticism. In our experience, all of our patients accepting help have been adequately vaccinated with SARS-CoV-2 vaccines without any substantial problems. 

## Acknowledgment 

We thank Vera Mahler for helpful comments and corrections. 

## Funding 

This project was funded by the Bavarian State Ministry of Science and Art, file number H.4001.1.7-53-7-TUME-FME-DE0BK. 

## Conflict of interest 

The authors declare no conflict of interest. 

**Table 1.** Lessons on SARS-CoV-2 and allergy in recent years.

Skin reactions associated with SARS-CoV-2 infection	Common maculopapular exanthema, papulovesicular exanthema, acute urticaria, and vascular reactions
Hypersensitivity reactions to drugs used to control SARS-CoV-2 infection	Rare, no typical triggers of hypersensitivity reactions
Side effect rate of SARS-CoV-2 vaccination	Low and overall comparable to other vaccines
Common general adverse reactions to SARS-CoV-2 vaccination	As with other vaccines, predominantly flu-like symptoms
Skin reactions after SARS-CoV-2 vaccination	In particular, local reactions, urticaria and morbilliform exanthema, also worsening of chronic inflammatory skin diseases, pityriasis rosea and zoster. Recurrence with follow-up vaccination < 50%.
Severe complications after SARS-CoV-2 vaccination mRNA vaccines	Increased risk of myocarditis and pericarditis, mostly mild and no contraindication for follow-up vaccination after healing. Vector vaccines: thrombosis at atypical sites especially in younger patients, and questionable Guillain-Barré syndrome, both contraindications for these vaccines
Risk of anaphylaxis to vaccination	Initially many individual cases of anaphylaxis reported, significantly increased risk of anaphylaxis feared, incidence now equal to or not substantially above that of other vaccines. Frequently women, allergies often reported, previous anaphylaxis
Vaccination in “allergic” patients	Procedure according to flow chart (Figure 2)
Vaccination in patients with contact sensitization to PEG	No evidence of PEG immediate type allergy; we have advised patients to vaccinate and have several reports of good tolerability
Allergy testing prior to vaccination	In Germany/Europe if there is evidence of vaccine hypersensitivity or PEG allergy. In the U.S., increasingly vaccination without prior allergy testing on an emergency standby basis
Allergy testing methods	Depending on availability and experience, skin prick test, intradermal test if necessary, basophil activation test and provocation tests with vaccine residues and adjuvants.
Vaccination of PEG-allergic patients	Possible after allergy diagnostics, after appropriate documented information and consent of the patient under appropriate monitoring and emergency preparedness.
